# Genome of the Southern Giant Petrel Assembled Using Third-Generation DNA Sequencing and Linked Reads Reveals Evolutionary Traits of Southern Avian

**DOI:** 10.3390/ani11072046

**Published:** 2021-07-09

**Authors:** Sun-Hee Kim, Seung-Jae Lee, Euna Jo, Jangyeon Kim, Jong-U Kim, Jeong-Hoon Kim, Hyun Park, Young-Min Chi

**Affiliations:** 1 Department of Biotechnology, College of Life Sciences and Biotechnology, Korea University, Seoul 02841, Korea; choihjdy@gmail.com (S.-H.K.); skullcap@korea.ac.kr (S.-J.L.); eunajo@kopri.re.kr (E.J.); bryan0413@naver.com (J.K.); 2 Greenwitch Co., 20, Jeungpyeong 2 Sandan-ro, Doan-myeon, Jeungpyeong-gun 27902, Korea; 3 Division of Life Sciences, Korea Polar Research Institute (KOPRI), Yeonsu-gu, Incheon 21990, Korea; sgj964970@gmail.com

**Keywords:** southern giant petrel, *Macronectes giganteus*, genome assembly, annotation, PacBio sequencing, 10x Genomics Chromium technology

## Abstract

**Simple Summary:**

The southern giant petrel *Macronectes giganteus* is one of key species on the Antarctic and sub-Antarctic regions. We reported the whole genome of *M. giganteus* for understanding the evolutionary mechanisms on Antarctic environments and for studying a more effective genetic monitoring of threatened species. The *M. giganteus* genome was 1.248 Gb in size with a scaffold N50 length of 27.4 Mb and a longest scaffold length of 120.4 Mb, and its genome contains 14,993 predicted protein-coding genes. The estimated historical effective population size of southern giant petrel underwent a severe reduction during a period coinciding with the early Pleistocene. The *M. giganteus* showed genomic expansion related to maintenance of energy homeostasis, being essential for survival and effective functioning in cold environments. Moreover, we employed a classification of microsatellite markers for studying the genetic diversity within and among populations. Genomic research of this first Antarctic bird helps to address the environmental adaptation and evolution of avian species.

**Abstract:**

The southern giant petrel *Macronectes giganteus*, a large seabird of the southern oceans, is one of only two members of the genus *Macronectes* and is the largest species in the order Procellariiformes. Although these two families account for the vast majority of the avian fauna inhabiting the Antarctic and sub-Antarctic regions, studies on the status of some populations and the associated genetic data are currently extremely limited. In this study, we assembled the genome of *M. giganteus* by integrating Pacific Biosciences single-molecule real-time sequencing and the Chromium system developed by 10x Genomics. The final *M. giganteus* genome assembly was 1.248 Gb in size with a scaffold N50 length of 27.4 Mb and a longest scaffold length of 120.4 Mb. The *M. giganteus* genome contains 14,993 predicted protein-coding genes and has 11.06% repeat sequences. Estimated historical effective population size analysis indicated that the southern giant petrel underwent a severe reduction in effective population size during a period coinciding with the early Pleistocene. The availability of this newly sequenced genome will facilitate more effective genetic monitoring of threatened species. Furthermore, the genome will provide a valuable resource for gene functional studies and further comparative genomic studies on the life history and ecological traits of specific avian species.

## 1. Introduction

Giant petrels (*Macronectes* spp.) belonging to the family Procellariidae of the order Procellariiformes are pelagic birds distributed throughout the Southern Ocean and Antarctic region. Members of the genus *Macronectes* were initially considered to be single polymorphic species until Bourne and Warham [1] separated these into two sibling species based on morphological and behavioral differences: The southern giant petrel *Macronectes giganteus* and the northern giant petrel *Macronectes halli*. Nonetheless, as hybridization and back-crossing have been reported in some habitats where both species breed sympatrically, their reproductive isolation would appear to be incomplete [2]. Both species are categorized as species of “Least Concern” on the International Union for Conservation of Nature (IUCN) Red List of Threatened Species 2018 [3].

With a wingspan of 150–210 cm and a body mass of 3.8–5.0 kg, the southern giant petrel is the largest species in the order Procellariiformes, [4], and two representative color morphs, characterized by grayish-brown and white with scattered blackish plumage, are recognized. Moreover, adults display sexual size dimorphism, with the males having larger bills and wing lengths and being heavier than females [5]. Hunter [6] suggested that sexual dimorphism may represent an adaptation associated with differences in foraging behavior with respect to preferred prey items, namely, krill and fish versus penguin carcasses foraged by females and males, respectively. Unlike other piscivorous petrels, the southern giant petrel feeds on animal carcasses [7], garbage from fishing vessels, and marine organisms such as fish, krill, and cephalopods [3]. These petrels can convert their high-fat diets into stomach oil comprising wax esters and triglycerides to feed their chicks, and which can be used as an energy source for long-distance flights [8]. Moreover, given that this stomach oil is not only sticky, but also gives off a sickening odor, the birds use the oil as a defensive weapon against intruders approaching their nest by spraying this from their bills. The bills of these birds are also characterized by long nasal tubes connected to salt glands located on the upper mandible, which play a role in excreting salt from the body [8].

Technical advances in the past decade have improved access to sequencing data, with lower costs leading to an explosion in the number of species. Genomes from many diverse organisms have been sequenced of high-quality references using hybrid approaches that combine complementary technologies, e.g., PacBio, 10x Genomics, and Hi-C sequencing technologies. Despite the increasing availability of genetic resources with research and economic value, a fully annotated genome is currently limited for Antarctic avian. Genomic resources has proven itself invaluable, not only for informing the understanding of the environmental adaptations, but for illuminating evolutionary mechanisms and forces. In Antarctic birds, large-scale genome analysis in 18 penguin species has shown that changing climatic conditions, i.e., changes in thermal niches, are accompanied by adaptations in genes that govern thermoregulation and oxygen metabolism, largely leading to the lineage diversification of the species [9]. Herein, we report the genome of *M. giganteus* assembled by integrating Pacific Biosciences single-molecule real-time sequencing and the Chromium system developed by 10x Genomics. We subsequently estimated the phylogenetic position of *M. giganteus* relative to that of other avian species and examined its historical effective population size. The genome of the southern giant petrel will facilitate more effective genetic monitoring of threatened species, thereby enabling us to conserve species based on a more comprehensive understanding of their evolutionary mechanisms. Moreover, it will provide a basis for gene functional studies and further comparative genomic studies on the life history and ecological traits of specific avian species.

## 2. Materials and Methods

### 2.1. Sample Collection

**Figure 1:**
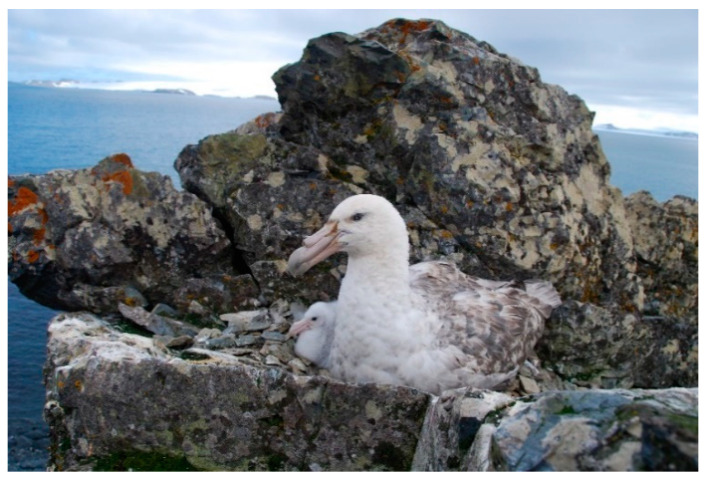
The southern giant petrel *Macronectes giganteus*.

Blood samples (approximately 5 mL) were collected from a male southern giant petrel (Figure 1) captured on King George Island, South Shetland Islands, Antarctica. These samples were immediately stored at −20 °C and subsequently used to extract high molecular weight genomic DNA (gDNA) using a MagAttract HMW DNA Kit (QIAGEN, Germantown, MD, USA) according to the manufacturer’s protocol. The quality and quantity of the isolated gDNA were determined using a 5400 Fragment analyzer (Agilent Technologies, Palo Alto, CA, USA) and a Qubit 2.0 Fluorometer (Invitrogen, Life Technologies, Carlsbad, CA, USA).

### 2.2. Library Construction and Sequencing

Genomic DNA libraries were prepared according to the manufacturer’s instructions. A single-molecule real-time sequencing bell (SMRT Bell) library was produced using a PacBio DNA Template Prep Kit 1.0 (Pacific Biosciences, Menlo Park, CA, USA). The quality and quantity of each library were assessed using a 2100 Bioanalyzer (Agilent Technologies). A SMRT Bell-Polymerase Complex was produced using a PacBio Binding Kit 2.0 (Pacific Biosciences) according to the manufacturer’s instructions. The complex was loaded into SMRT cells (Pacific Biosciences, Sequel™ SMRT^®^ Cell 1M v2) and sequenced using a Sequel Sequencing Kit 2.1 (Pacific Biosciences) in conjunction with P6-C4 chemistry. For each SMRT cell, 1 × 600 min movies were captured using the Sequel sequencing platform (Pacific Biosciences) at DNA Link (Seoul, Korea).

A Chromium sequencing library for the southern giant petrel was generated using 10x Genomics Chromium technology according to the manufacturer’s instructions. Gel beads-in-emulsion (GEMs) were generated from a library of genome gel beads combined with 1.5 ng of gDNA in a master mix and partitioning oil, using a 10x Genomics Chromium Controller instrument in conjunction with a micro-fluidic Genome chip (PN-120257). The GEMs were then subjected to isothermal incubation. Barcoded DNA fragments were extracted and subjected to Illumina library construction, as detailed in the Chromium Genome Reagent Kits Version 2 User Guide (PN-120258). The library yield was measured using a Qubit dsDNA HS Assay Kit (Thermo Fisher Scientific, Waltham, MA, USA) and library fragment size and distribution were determined using an Agilent 2100 Bioanalyzer High Sensitivity DNA chip (Santa Clara, CA, USA). The DNA was sequenced using a NovaSeq sequencer with a 2 × 150 bp read metric, generating approximately 1.6 billion paired-end (PE) reads (Table S1).

### 2.3. Genome Assembly

The size of the genome was estimated using Jellyfish 2.1.4 [10] with a K-mer value of 25. For estimations of genome size, heterozygosity rate, and repeat content, we utilized GenomeScope [11] in R (version 3.4.4) [12]. A Falcon-Unzip assembler (version 0.4; Falcon, RRID:SCR_016089) was used for de novo assembly [13], with the options length_cutoff = 12,000 and length_cutoff_pr = 10,000. We polished the initial genome assembly in order to improve its accuracy with Pilon v1.22 (Pilon, RRID:SCR_014731) [14], using Bowtie v2.3.4.1 [15] with the short-read assembly dataset, and we used Purge Haplotigs [16] to detect and remove haplotigs from the assembly. Using the initial assemblies thus obtained, we conducted scaffolding using the Assembly Roundup by Chromium Scaffolding (ARCS) algorithm, a method that leverages the rich information content of high-volume long sequencing fragments to further organize draft genome sequences into contiguous assemblies [17].

To evaluate the completeness of the southern giant petrel genome assembly, the assembled *M. giganteus* scaffolds were subjected to analysis using Benchmarking Universal Single-Copy Orthologs (BUSCO) version 4.0 (RRID:SCR 015008) with default parameters by the conservation of a core set of genes in the Aves database (aves_odb9) [18].

### 2.4. Gene Prediction and Annotation

A *de novo* repeat library was constructed using RepeatModeler version 1.0.3 (RRID:SCR 015027) [19], RECON version 1.08 [20], and RepeatScout version 1.0.5 (RRID:SCR 014653) [20], with default parameters. Tandem Repeats Finder was used to predict consensus sequences, classification information for each repeat, and tandem repeats, including simple repeats, satellites, and low-complexity repeats [21]. Using the de novo repeat library, repetitive elements were identified using RepeatMasker (version 4.0.9, RRID:SCR_012954), and the repeat landscape was calculated using the Kimura distance for each alignment.

Genome annotation was performed using MAKER version 3.01 (MAKER, RRID:SCR 005309), which is a portable and readily configurable genome annotation pipeline [22]. Subsequently, repeat masked genomes were used for ab initio gene prediction using SNAP v2006–07-28 [23] and Augustus (version 3.2.3, RRID:SCR_008417) [24]. MAKER was initially run in the est2genome mode based on *M. giganteus* transcriptome sequencing data [25]. In the second MAKER run, protein sequences from 25 avian species (Table S2) were used as protein evidence data. During this run, we used different est2 genome and protein 2 genome settings, whereas all other settings were the same as those used for the initial run. Subsequently, following retraining, MAKER selected and revised the final genome model. To select the best-supported gene models, we used the quality metric annotation edit distance (AED), developed by Sequence Ontology [26]. More than 90% of the annotations were found to have AEDs of less than 0.5 [27]. The final gene set of the protein-coding genes was thus screened using an AED < 0.5.

The predicted genes were annotated by alignment against the NCBI non-redundant protein database using BLASTP version 2.2.29 [28] with a maximum e-value of 1e-5. Protein signatures were annotated using InterProScan 5 (version 5.44.79; RRID:SCR_005829), [29] and Gene Ontology (GO) terms were assigned using the BLAST2GO pipeline (version 4.1.9) [30]. Pathway annotation analysis used the Kyoto Encyclopedia of Genes and Genomes (KEGG) Automatic Annotation Server.

### 2.5. Comparative Genomics

For comparisons of genome sequences at the chromosome level, we used MUMmer (version 4.02b, RRID:SCR_018171) [31]. This was used to compute the raw sequence hits between two genomes with a minimum alignment length of 300 bp. Circos (RRID:SCR_011798) [32] was used to compare genome sequences based on the homogeneous coordinates identified using MUMmer.

### 2.6. Population History

The historical effective population size (Ne) was determined using the pairwise sequential Markovian coalescent (PSMC) model with the following settings: –N25, –t15, –r5, –p “4 + 25 × 2 + 4 + 6” [33]. The neutral mutation rate was estimated using the divergence time, and sequence divergence based on comparisons between Northern fulmar and Southern giant petrel genomes shotgun data was estimated using the LASTZ [34] with setting (–step = 19–hspthresh = 2200–inner = 2000–ydrop = 3400–gappedthresh = 10,000–format = axt). The generation time was assumed to be seven years for each species [35]. To determine the variance in Ne estimates, we performed 100 bootstraps for each species.

### 2.7. Gene Family Identification and Phylogenetic Analysis

Orthologous gene clusters of the annotated genomes of the 26 assessed species (Table S2) were classified using the OrthoMCL pipeline [36] by applying the Markov clustering algorithm (MCL) [37] with default parameters. A phylogenetic tree was constructed based on a single-copy orthologous group of genes common to all 26 avian species. A Probabilistic Alignment Kit (PRANK) [38] was used to align protein-coding genes with the codon alignment option. Gblocks [39] was used to eliminate poorly aligned regions with gaps and less than 40% identity, as well as genes shorter than 200 bp, using a codon model for the subsequent procedures. A maximum-likelihood tree was constructed using RAxML (RRID:SCR 006086) [40] with 1000 bootstrap replicates. Divergence time calibration was performed using TimeTree (with minimum and maximum divergence times for *Calypte anna* and *Chaetura pelagica* of 52 and 62; *Geospiza fortis* and *Taeniopygia guttata*, 30.4 and 46.8; and *Pygoscelis adeliae* and *Aptenodytes forsteri*, 12.6, and 33.3 million years ago (Mya), respectively) [41] using MEGA X [42].

### 2.8. Simple Sequence Repeat Identification

Simple sequence repeat (SSR) loci within the *M. giganteus* genome sequence were identified using QDD 3.1 [43]. Five types of SSR (di-, tri-, tetra-, penta-, and hexa-nucleotide) motifs were extracted, and the SSR loci in the genic region were filtered using the gene annotation results to select the more conserved markers.

## 3. Results

### 3.1. Genome Assembly

From eight PacBio sequel cells, we generated 6,117,480 error-corrected reads with a mean length of 10.9 kb. The total coverage of the PacBio reads was computed using 1.3 Gbp of the genome size estimated from the K-mer value (Figure S1). The PacBio error-corrected long reads were initially assembled using Falcon-Unzip to generate a draft assembly spanning 1248 Mb, with a contig N50 length of 6.27 Mb and a longest contig length of 20.83 Mb (Table 1). To achieve higher contiguity, we applied the 10x Genomics Chromium platform, an integrated solution that can be used to generate short Illumina reads with long-range positional information. We sequenced 823 million reads comprising 124 Gb of the Chromium 10x Genomics Linked-read libraries (Table S1). Further scaffolding procedures were performed using ARCS to obtain the final consensus genome sequence. The size of the scaffold assembly was 1.247 Gb (94% of the predicted 1.328 Gb genome size) (Table 1).

BUSCOs were identified in the assembled genome as complete BUSCO profiles within the avian clade ortholog gene databases, aves_odb9 dataset of 4627, which contain 4915 genes. Of these, 4570 (93.0%) were single-copy and 57 (1.2%) were duplicated BUSCOs. The numbers of partially matched and missing BUSCOs were 183 (3.7%) and 105 (2.1%), respectively. A total of 97.9% of the BUSCO genes were identified in the *M. giganteus* genome (Table 2).

**Figure 2:**
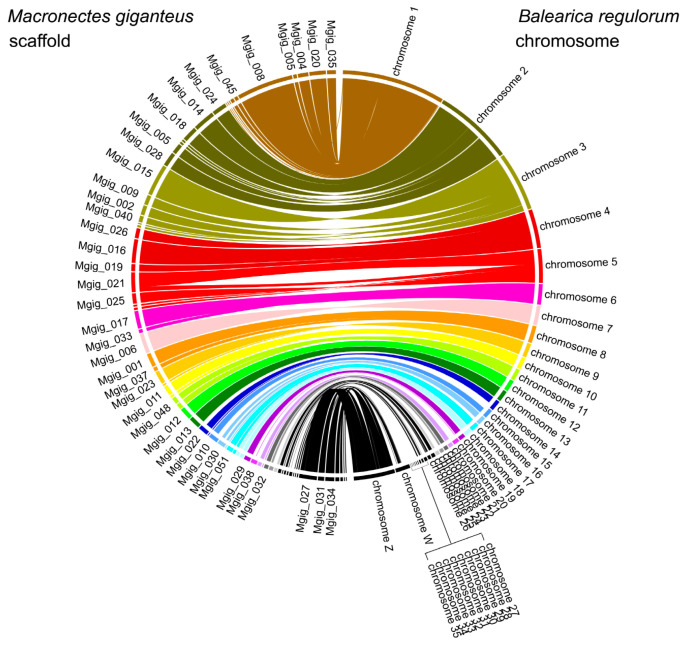
Collinear relationship between *Macronectes giganteus* and *Balearica regulorum*. Lines between the two rectangles show the shared syntenic blocks between the chromosomes, based on sequence homology.

To confirm the genome stability of the constructed *M. giganteus* genome, we compared our data to those pertaining to the *Balearica regulorum* (gray crowned crane) genome. A total of 1.191 Gb (>1 Mb) of the assembled *M. giganteus* genome was mapped to that of *B. regulorum*. However, whereas 28 chromosomes and the Z chromosome of *B. regulorum* were highly contiguous with those of *M. giganteus* (Figure 2), chromosomes 29 to 35, which are small-sized chromosomes (6.9–1.2 Mb) in *B. regulorum* were not mapped.

The *M. giganteus* genome contains 11.06% repeat sequences, of which 8.61% comprises transposable elements (TEs), including long interspersed nuclear elements (LINEs) (3.06%), short interspersed nuclear elements (SINEs) (0.07%), long terminal repeats (LTRs) (5.45%), and DNA transposons (0.34%). Notable among these repeats are the ERVL and gypsy LTRs and CR1 LINE, which were detected in the majority of the *M. giganteus* genome (Table S3).

### 3.2. Gene Prediction and Annotation

A total of 14,993 protein-coding genes were annotated in the *M. giganteus* genome by combining the results of gene prediction, homology searches, and transcriptional evidence. These genes contained an average of 10.1 exons per gene, comprising a total exon length of 27.6 Mb (Table 3). The predicted genes were initially annotated by alignment with the NCBI non-redundant protein (nr) database [44] using NCBI BLAST, resulting in the annotation of 14,779 genes. To obtain protein domain information, we annotated 14,764, 12,665, and 10,033 genes using the InterProScan, EggNOG, and Pfam databases, respectively. Consequently, 14,822 genes were annotated in one or more databases, which represents 98.86% of the total genes (Table 3). GO category functional classification was performed using BLAST2GO v5.25, which enabled us to annotate 12,562 genes. Similarly, pathway annotation analysis assigned 11,575 genes using the KEGG database (Table 3).

### 3.3. Population History and Gene Phylogenomics

**Figure 3:**
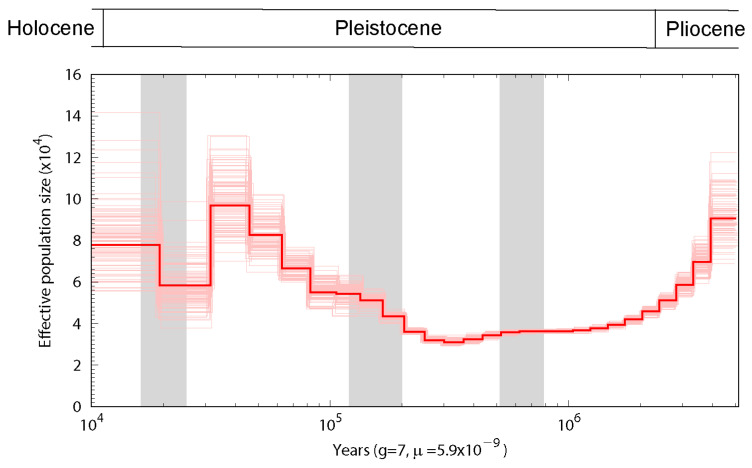
Inference of southern giant petrel population history based on pairwise sequential Markovian coalescent (PSMC) analysis. The red curve is the PSMC estimate for the original data, and the pink curves indicate the PSMC estimates for 100 bootstrapped sequences. The last glacial maximum (LGM), penultimate glaciation and the Naynayxungla glaciation are highlighted in gray vertical bars.

PSMC analysis [33] based on a hidden Markov model has been used to estimate the history of effective population size (Ne) based on genome-wide heterozygous sequence data, and in the present study, analysis using the PSMC model revealed evidence of population expansion from 200 to 300 thousand years ago, followed by a subsequent contraction in size (Figure 3).

**Figure 4:**
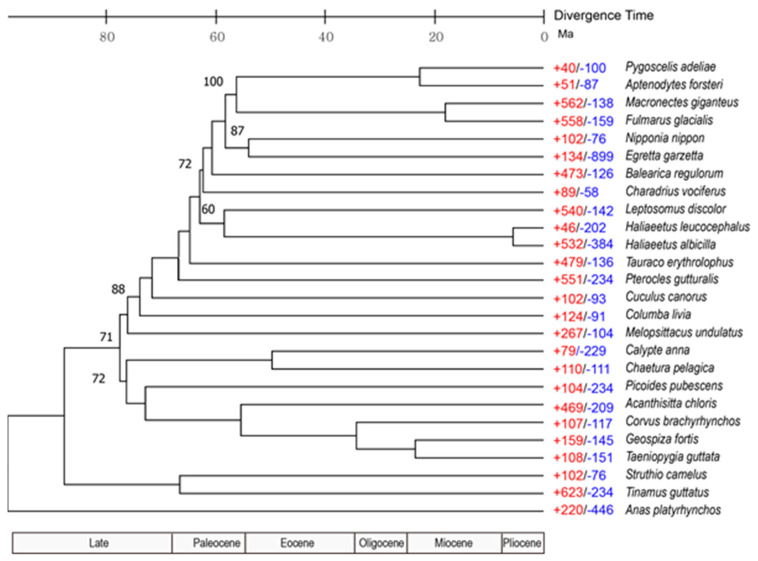
Phylogenetic tree and gene family gain-and-loss analysis, including the number of gained (+) and lost (−) gene families. All bootstrap values are 100%, except where denoted.

We used OrthoMCLv1.2 [36] to cluster gene families, with proteins from 25 known avian genomes and *M. giganteus* being all-to-all blasted using BLASTP with an e-value threshold of 1e-5, and for genes with alternative isoforms, we used only proteins derived from the longest transcript. We identified 11,628 gene families in the genomes of *M. giganteus* and other avian species. Among the resulting orthogroups, 6862 orthologous gene families were commonly identified in the 26 avian genomes, including 3405 single-copy ortholog groups and 208 multi-copy ortholog groups. The southern giant petrel genome was found to have 1679 paralogous groups containing 4459 genes, of which 28 comprising 98 genes were *M. giganteus*-specific (Figure S2). Using single-copy orthologs, we were able to probe the phylogenetic relationships of *M. giganteus* and other avian species to examine evolutionary relationships using RAxML software with maximum-likelihood genome-wide phylogenetic analysis (Figure 4).

**Figure 5:**
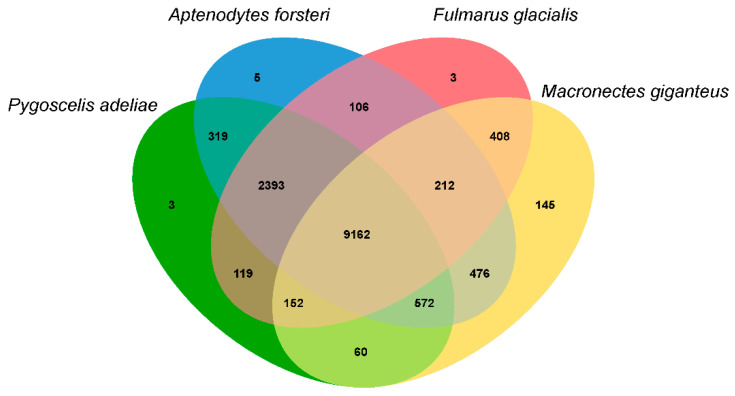
A Venn diagram of the paralogous and orthologous groups among the Adélie penguin (*Pygoscelis adeliae*), emperor penguin (*Aptenodytes forsteri*), northern fulmar (*Fulmarus glacialis*), and southern giant petrel (*Macronectes giganteus*) obtained using OrthoFinder.

Analyses of gene gain and loss among genomes facilitate the reconciliation of species trees with the gene tree for each family, and using this approach, we established that the southern giant petrel has 562 significantly expanded and 138 significantly contracted gene families (Figure 4 and Tables S4 and S5). In terms of the GO category biological process, we found that the vast majority of expanded pathways are associated with protein catabolism, including proteolysis involved in the cellular protein catabolic process (GO:0051603), the modification-dependent protein catabolic process (GO:0019941), and the ubiquitin-dependent protein catabolic process (GO:0006511). Among the four species we identified as being phylogenetically closely related (Adélie penguin, emperor penguin, northern fulmar, and southern giant petrel), we detected 9162 common gene families (Figure 5). These findings thereby indicate that these species have close genetic relationships. A total of 145 gene families, comprising 566 genes, were found to be specific to *M. giganteus*, and are typically associated with five GO biological pathways, including smoothened signaling pathways involved in the regulation of secondary heart field cardioblast proliferation (GO:0003271) related to heart development and the ubiquitin-dependent protein catabolic process (GO:0006511) (Table S6).

### 3.4. Microsatellite Marker Identification

**Figure 6:**
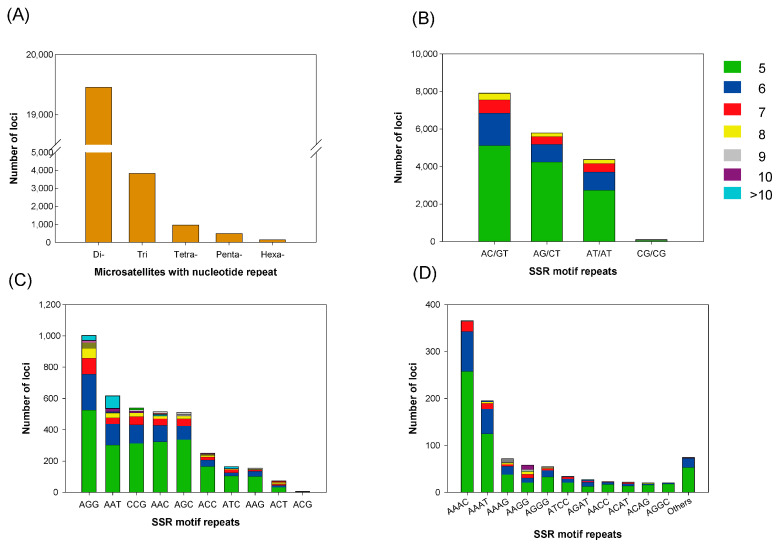
The distribution and frequency of microsatellite motifs. (**A**) Frequency of different microsatellite repeat types. (**B**–**D**) Frequency of different dinucleotide, trinucleotide, and tetranucleotide microsatellite motifs, respectively. The colors for (**B**–**D**) indicate the frequency of microsatellite mofits.

Microsatellite markers, also referred to as simple sequence repeats (SSRs), are routinely employed for estimating genetic diversity in population genetics studies and are often implicitly assumed to reflect the genome-wide diversity of a taxon [45]. In the present study, we identified a total of 24,887 SSR loci within the southern giant petrel genome (Figure 6 and Table S7), among which, dinucleotides (19,456) accounted for 78.18%, and of these, 75.08% have AC/GT or AG/CT motifs, whereas the those with a CG/CG motif accounted for a mere 0.59% of the total SSR loci. Of the remaining SSR loci, trinucleotides (3830), tetranucleotides (966), pentanucleotides (495), and hexanucleotides (140) accounted for 15.40%, 3.88%, 1.99%, and 0.56%, respectively.

## 4. Discussion

Recently, reference-mapped genome assembly of the southern giant petrel using short-read Illumina data have been reported [9], though the genome was fragmented into many scaffolds due to low coverage and sequencing by synthesis technology. With the development of third-generation single-molecule sequencing technology, long-read sequences can be precisely assembled into genome and discovery features of DNA areas that have previously unavailable DNA regions. The genome of *M. giganteus* was assembled using the PacBio long reads and 10x Genomics Chromium platform, achieving a final scaffold assembly of 1.247 Gb (94% of the predicted 1.328 Gb genome size). The final scaffolding assembly resulted in a significantly improved assembly, the longest scaffold length was 120 Mb, and the scaffold N50 value was 27 Mb, which were 6- and 4.5-fold greater than the corresponding draft assembly values, respectively (Table 1). Thirty-six superscaffolds were greater than 10 Mb in size, and 74 scaffolds were over 1 Mb in size. The total assembly length of over 1 Mb was 1.191 Gb.

We compared our data to those pertaining to the *B. regulorum* genome to confirm the chromosomal stability of the constructed *M. giganteus* genome. Chromosome 1 of *B. regulorum* was found to be highly contiguous with the Mgig_0035, Mgig_0020, Mgig_0004, Mgig_0055, Mgig_0008, Mgig_0045, and Mgig_0043 scaffolds of *M. giganteus*, with small portions of syntenic inversion. However, these seven *M. giganteus* scaffolds mapped two chromosomes (chromosomes 1 and 4) of *Tauraco erythrolophus* (Figure S3). Although the southern giant petrel genome sequence does not fully assemble at the chromosome level, lineage-specific chromosome rearrangements are evident, as confirmed by analysis of the chromosomes of other species, despite the paucity of translocations during evolution.

The repeat sequences of *M. giganteus* genome contains 11.06% repeat sequences, of which 8.61% comprise transposable elements (TEs). In order to estimate the “relative age” and transposition history of TEs [46], Kimura distances (K-values) were calculated for all TE copies of each element. Copy divergence is correlated with the age of activity, with very similar copies (low K-values) being indicative of rather recent activity, whereas divergent copies (high K-values) are assumed to have been generated by more ancient transposition events. Kimura substitution levels indicated significant interspecific differences in profiles (Figure S4), with the *M. giganteus* genome being dominated by relatively recent copies (K-values <5) and strongly shaped by LINE and LTR transposons, which can be taken to be indicative of recent bursts of transposition in *M. giganteus*. TE expansion may have facilitated gene duplication and other genomic evolutionary events during particular periods of evolutionary history [47], and thus may have contributed to adaptation to the specifics of Antarctic environments.

A total of 14,993 protein-coding genes were annotated in the *M. giganteus* genome. The predicted genes were initially annotated by alignment with the NCBI nr database, and subsequently using the InterProScan, EggNOG, and Pfam databases. Consequently, 14,822 genes were annotated in one or more databases, which represents 98.86% of the total genes.

The estimated history of effective population size (Ne) using PSMC analysis revealed evidence of population expansion from 200 to 300 thousand years ago, followed by a subsequent contraction in size (Figure 3). The Ne curve shown in Figure 3 indicates a reduction during the last glacial maximum and population growth during the last glacial period. Historically, global climate change has substantially influenced the distribution and abundance of biodiversity, including that of birds. In particular, during unfavorable glacial periods, many species experienced range contractions, followed by subsequent expansions during interglacial periods [48]. A number of the species currently included in the IUCN Red List of Threatened Species have shown a long-term reduction in population size, predating recent declines [48], among which, the southern giant petrel experienced a long-term reduction in Ne to 40,000 individuals around 0.5 Mya.

Phylogenetic relationships of *M. giganteus* and other avian species to examine evolutionary relationships showed that clustering in the constructed phylogenetic tree indicates that the southern giant petrel and northern fulmar (*Fulmarus glacialis*) form a sister group, and that the order Sphenisciformes (including the Adélie penguin and emperor penguin) have a close genetic relationship. On the basis of phylogeny, we calculated species divergence times according to the molecular clock with respect to TimeTree, and accordingly estimated that *M. giganteus* and *F. glacialis* diverged 19.97 Mya.

The southern giant petrel has 562 significantly expanded gene families, and the vast majority of expanded pathways are associated with protein catabolism, including proteolysis involved in the cellular protein catabolic process (GO:0051603), the modification-dependent protein catabolic process (GO:0019941), and the ubiquitin-dependent protein catabolic process (GO:0006511). The southern giant petrel is circumpolar, with a distribution that encompasses the sub-Antarctic Islands and the Antarctic Peninsula [49]. Maintenance of energy homeostasis is essential for survival and effective functioning in cold environments, and intracellular energy homeostasis is closely related to protein degradation and synthesis, including the functioning of the ubiquitous-dependent protein and autophagy systems for protein decomposition and synthesis as energy-saving processes. Consequently, the expansion of these genes may be required to maintain efficient energy homeostasis in cold environments. Among the KEGG metabolic pathway maps we obtained, the MAPK signaling pathway of signal transduction was activated in response to almost any change in the extracellular or intracellular environment that affects the metabolism of cells, organs, or entire organisms required for physiological metabolic adaptation shown to be expanded. Genes associated with carbohydrate and lipid metabolism were also frequently found to be expanded (Figures S5 and S6).

The breeding populations of southern giant petrels are distributed on several sub-Antarctic islands, the Antarctic Peninsula, southern Chile, the Malvinas (Falkland) Islands, and Patagonia, Argentina [50]. Although the trends show a general decline in the total breeding population of *M. giganteus*, whereas some colonies have decreased in size over the past few decades, others have increased [49]. Such population declines are attributed to the detrimental effects of habitat destruction, human disturbance, introduced predators, and fisheries. In this regard, a sufficient genetic diversity is essential to enable adaptation to changing environmental conditions, and is recognized as a key component of biodiversity. *M. giganteus* and *M. halli* are the only members of the genus *Macronectes*. Although *M. giganteus* breed both further north and further south than *M. halli*, these two species breed sympatrically across five islands: South Georgia, the Prince Edward Islands, Îles Crozet, Îles Kerguelen, and Macquarie Island. The proportion of crossbred species pairs has been reported to be 0.4–2.4% annually for South Georgia island [1]. We identified a total of 24,887 SSR loci within the southern giant petrel genome. These microsatellite markers will provide useful information for future analyses of the genetic diversity within and among populations, and also these data can be used to identify hybrids between giant petrel species. The SSR data will, in turn, enable us to draw conclusions regarding genome-wide diversity patterns pertinent to conservation of the southern giant petrel, and potentially contribute to research on the population trends of other listed threatened species.

## 5. Conclusions

In this study, we assembled the genome of the southern giant petrel based on integrating Pacific Biosciences single-molecule real-time sequencing and the Chromium system developed by 10x Genomics. The annotated genome was found to contain 14,993 protein-coding genes. Moreover, analysis of the data obtained indicated that the southern giant petrel underwent a severe reduction in effective population size coinciding with the early Pleistocene period, with the potential for recovery during mild periods. The genome of the southern giant petrel will facilitate a more effective genetic monitoring of threatened species, which, by enhancing our understanding of evolutionary mechanisms, will enable us to conserve such species, and will also contribute to further genetic studies on the life history and ecological traits of avian species.

## Figures and Tables

**Table 1:** Summary of the *Macronectes giganteus* genome assembly.

	Contigs (Falcon-Unzip)	Scaffolds (ARCS)
Number of scaffolds	1199	861
Total size of scaffolds	1,247,954,784	1,247,958,164
Longest scaffold	20,833,995	120,319,780
Shortest scaffold	15,076	15,076
Number of scaffolds >10 M nt	21	36
N50 scaffold length	6,274,151	27,376,298
L50 scaffold number	61	14
G+C content (%)	42.76	42.76

**Table 2:** Assessment of the *Macronectes giganteus* genome assembly via BUSCO.

aves_odb9	No.	%
Complete BUSCOs (C)	4627	94.2
Complete and single-copy BUSCOs (S)	4570	93
Complete and duplicated BUSCOs (D)	57	1.2
Fragmented BUSCOs (F)	183	3.7
Missing BUSCOs (M)	105	2.1
Total BUSCO groups searched	4915	

**Table 3:** Summary of the *Macronectes giganteus* gene annotations.

	Value
Gene length sum (bp)	269,696,814
Gene count	14,993
Exon length sum (bp)	27,620,459
Exon count	152,053
CDS length sum (bp)	25,123,992
CDS count	151,205
NCBI-NR	14,779
Uniprot/Swiss-prot annotations	10,034
InterProScan annotations	14,764
Pfam annotations	10,033
EggNOG annotations	12,665
Gene Ontology annotations	12,562
KEGG annotations	9154

## Data Availability

Sequence data obtained in the southern giant petrel genome project were deposited in the NCBI database under the BioProject number PRJNA702664. The whole-genome sequence was deposited in the Sequence Read Archive (SRA) database under the accession numbers SRR13736509 and SRR13736510.

## Supplementary Materials

The following are available online at https://www.mdpi.com/article/10.3390/ani11072046/s1, Table S1. Summary of *Macronectes giganteus* genome sequencing; Table S2. The genome assemblies/gene models used in this study; Table S3. Summary of repetitive elements in the *Macronectes giganteus* genome; Table S4. Gene Ontology of expanded gene families in the *Macronectes giganteus* genome relative to that of 25 avian species; Table S5. Gene Ontology of the contracted gene families in the *Macronectes giganteus* genome relative to that of 26 avian species; Table S6. Enrichment Gene Ontology for the *Macronectes giganteus*-specific gene families among four avian species; Table S7. Statistics of *Macronectes giganteus* microsatellites; Figure S1. A histogram of the 25-mer depth distribution was plotted in GenomeScope to estimate the genome size (1328 Mb) and the heterozygosity level (0.115%); Figure S2. Comparison of avian orthologous genes. A comparative representation of orthologous and paralogous genes in 26 avian genomes are shown; Figure S3. (A) Collinear relationship between *Macronectes giganteus* and *Tauraco erythrolophus*. Lines between the two rectangles indicate the shared syntenic blocks between the chromosomes, based on sequence homology. (B) Chromosome rearrangements between *Macronectes giganteus*, *Balearica regulorum*, and *Tauraco erythrolophus*; Figure S4. Interspersed repeat landscape of (A) the *Macronectes giganteus* genome and (B) the interspersed repeat landscape of the *Gallus gallus* (galGal4), *Taeniopygia guttata* (taeGut1), and *Anas platyrhynchos* (anaPla1) genomes. Data from http://www.repeatmasker.org; Figure S5. (A) KEGG pathways of the expanded genes and (B) number of genes in the signal transduction pathway; Figure S6. Expanded genes of the glycerolipid metabolism in KEGG pathways. The red color indicates expanded genes.
