# Supplementary material for: Genome of the Southern Giant Petrel Assembled Using Third-Generation DNA Sequencing and Linked Reads Reveals Evolutionary Traits of Southern Avian
Source: Animals (Basel). 2021 Jul 9;11(7):2046. doi: 10.3390/ani11072046 (PMC8300169; doi:10.3390/ani11072046)
Supplement: Supplementary file 1 [file animals-11-02046-s001.zip › animals-1264316-supplementary.pdf]

Table S1. Summary of the *Macronectes giganteus* genome sequencing.

Sample	Library	No. of Cells	No. of Reads	Total data (Mbp)
DNA	Pacbio	8	6,117,480	66,762
	Linked read			
	GP_R1		426,268,891	64,366
	GP_R1		397,908,199	60,084

Table S2. Genome assemblies/Gene models used in this study.

Common name	Scientific name	Assembly	Assembly status
Southern giant petrel	*Macronectes giganteus*	This study	
Adélie penguin	*Pygoscelis adeliae*	GCF_000699105.1	Draft
American crow	*Corvus brachyrhynchos*	GCF_000691975.1	Draft
Anna's hummingbird	*Calypte anna*	GCF_003957555.1	Chromosome
Bald eagle	*Haliaeetus leucocephalus*	GCF_000737465.1	Draft
Budgerigar	*Melopsittacus undulatus*	GCF_012275295.1	Chromosome
Chimney swift	*Chaetura pelagica*	GCF_000747805.1	Draft
Common cuckoo	*Cuculus canorus*	GCF_000709325.1	Draft
Common ostrich	*Struthio camelus*	GCF_000698965.1	Draft
Crested ibis	*Nipponia nippon*	GCF_000708225.1	Draft
Cuckoo roller	*Leptosomus discolor*	GCF_000691785.1	Draft
Downy woodpecker	*Picoides pubescens*	GCF_000699005.1	Draft
Emperor penguin	*Aptenodytes forsteri*	GCF_000699145.1	Draft
Grey crowned crane	*Balearica regulorum*	GCF_000709895.1	Chromosome
Killdeer	*Charadrius vociferus*	GCF_000708025.1	Draft
Little egret	*Egretta garzetta*	http://gigadb.org/dataset/101002	Draft
Medium ground finch	*Geospiza fortis*	GCF_000277835.1	Draft
Northern fulmar	*Fulmarus glacialis*	GCF_000690835.1	Draft
Peking duck	*Anas platyrhynchos*	GCF_003850225.1	Chromosome
Pigeon	*Columba livia*	GCF_000337935.1	Draft
Red crested turaco	*Tauraco erythrolophus*	GCF_000709365.1	Chromosome
Rifleman	*Acanthisitta chloris*	GCF_000695815.1	Draft
White tailed eagle	*Haliaeetus albicilla*	GCF_000691405.1	Draft
White throated tinamou	*Tinamus guttatus*	GCF_000705375.1	Draft
Yellow thoated sandgrouse	*Pterocles gutturalis*	GCF_000699245.1	Draft
Zebra finch	*Taeniopygia guttata*	GCF_008822105.2	Chromosome

Table S3. Summary of repetitive elements of *Macronectes giganteus*.

Class		Number of elements	Length (bp)	Percentage of sequence (%)
DNA		5,853	1,215,766	0.10
	CMC-EnSpm	3,534	275,257	0.02
	Crypton	111	13,900	0.00
	Crypton-A	204	25,697	0.00
	Kolobok	534	61,701	0.00
	MULE-MuDR	202	41,958	0.00
	Merlin	209	20,748	0.00
	PIF-Harbinger	6,677	586,421	0.05
	Sola-2	524	1,049,608	0.08
	TcMar	227	36,738	0.00
	TcMar-Tc1	728	176,382	0.01
	TcMar-Tigger	209	30,998	0.00
	hAT	1,573	205,790	0.02
	hAT-Ac	321	41,121	0.00
	hAT-Charlie	1,005	145,116	0.01
	hAT-Tag1	587	145,113	0.01
	hAT-Tip100	430	47,884	0.00
	hAT-hAT6	694	63,686	0.01
LINE				
	CR1	111,392	36,479,868	2.92
	L1	251	68,389	0.01
	L2	2,393	423,054	0.03
	Penelope	2,923	314,057	0.03
	RTE-BovB	570	100,102	0.01
LTR		143	88,807	0.01
	Copia	343	419,280	0.03
	DIRS	126	15,385	0.00
	ERV	62	39,052	0.00
	ERV1	2,528	737,962	0.06
	ERV4	1,982	594,888	0.05
	ERVK	579	451,769	0.04
	ERVL	27,589	18,510,661	1.48
	Gypsy	21,179	12,082,479	0.97
	Viper	20	20,707	0.00
	unknown	55,062	32,265,365	2.59
SINE				
	5S-Deu-L2	793	109,658	0.01
	MIR	4,751	583,102	0.05
	tRNA	1,039	115,129	0.01
	tRNA-Deu-L2	241	20,110	0.00
Unknown		56,081	17,528,143	1.40
	Total interspersed	313,669	125,151,851	10.03
Low_complexity		49,051	2,456,139	0.20
Satellite		261	29,257	0.00
Simple_repeat		248,761	9,801,849	0.79
rRNA		450	509,166	0.04
tRNA		55	4,220	0.00
Total		612,247	137,952,482	11.06

Table S4. Gene Ontology of expanded genes families in the *Macronectes giganteus* genome relative to the 26 species.

GO type	GO ID	GO Name	P-Value	Nr Test
BP	GO:0051603	proteolysis involved in cellular protein catabolic process	6.30E-09	81
BP	GO:0043632	modification-dependent macromolecule catabolic process	6.20E-09	77
BP	GO:0019941	modification-dependent protein catabolic process	2.30E-09	77
BP	GO:0006511	ubiquitin-dependent protein catabolic process	1.80E-09	77
BP	GO:0070646	protein modification by small protein removal	1.30E-16	54
BP	GO:0016579	protein deubiquitination	4.30E-16	51
BP	GO:0090132	epithelium migration	5.60E-07	31
BP	GO:0010631	epithelial cell migration	3.70E-07	31
BP	GO:0043542	endothelial cell migration	8.60E-07	27
BP	GO:0046148	pigment biosynthetic process	3.70E-08	19
BP	GO:0006582	melanin metabolic process	5.70E-12	19
BP	GO:0017015	regulation of transforming growth factor beta receptor signaling pathway	1.00E-06	19
BP	GO:0042438	melanin biosynthetic process	2.40E-12	19
BP	GO:1903844	regulation of cellular response to transforming growth factor beta stimulus	1.00E-06	19
BP	GO:0030511	positive regulation of transforming growth factor beta receptor signaling pathway	4.10E-11	17
BP	GO:0032435	negative regulation of proteasomal ubiquitin-dependent protein catabolic process	2.40E-09	16
BP	GO:0015800	acidic amino acid transport	5.00E-07	16
CC	GO:0005938	cell cortex	7.10E-05	27
CC	GO:0043198	dendritic shaft	9.30E-13	18
CC	GO:0043197	dendritic spine	1.70E-04	18
CC	GO:0044309	neuron spine	2.10E-04	18
CC	GO:0098862	cluster of actin-based cell projections	7.00E-05	15
CC	GO:0031588	nucleotide-activated protein kinase complex	7.80E-10	11
MF	GO:0140096	catalytic activity, acting on a protein	6.00E-05	232
MF	GO:0003677	DNA binding	6.50E-05	159
MF	GO:0008233	peptidase activity	1.10E-06	95
MF	GO:0030695	GTPase regulator activity	4.00E-05	58
MF	GO:0008234	cysteine-type peptidase activity	9.80E-16	58
MF	GO:0008242	omega peptidase activity	9.50E-20	52
MF	GO:0019783	ubiquitin-like protein-specific protease activity	2.60E-20	52
MF	GO:0004843	thiol-dependent deubiquitinase	4.30E-21	52
MF	GO:0101005	deubiquitinase activity	6.80E-21	52
MF	GO:0022832	voltage-gated channel activity	7.00E-05	29
MF	GO:0022843	voltage-gated cation channel activity	4.50E-06	26
MF	GO:0005267	potassium channel activity	9.20E-06	23
MF	GO:0005249	voltage-gated potassium channel activity	4.40E-05	17
MF	GO:0051213	dioxygenase activity	2.40E-04	15
MF	GO:0016706	2-oxoglutarate-dependent dioxygenase activity	7.90E-05	12
MF	GO:0030165	PDZ domain binding	5.00E-05	11
MF	GO:0004679	AMP-activated protein kinase activity	8.20E-08	10
MF	GO:0016307	phosphatidylinositol phosphate kinase activity	1.50E-06	9
MF	GO:0030552	cAMP binding	2.60E-05	7
MF	GO:0004065	arylsulfatase activity	2.60E-05	6
MF	GO:0005042	netrin receptor activity	7.20E-05	6
MF	GO:0005007	fibroblast growth factor-activated receptor activity	1.90E-04	5
MF	GO:0060072	large conductance calcium-activated potassium channel activity	1.10E-04	4
MF	GO:0030144	alpha-1,6-mannosylglycoprotein 6-beta-N-acetylglucosaminyltransferase activity	1.10E-04	4

Table S5. Gene Ontology of contracted genes families in the *Macronectes giganteus* genome relative to the 26 species.

GO type	GO ID	GO Name	P-Value	Nr Test
BP	GO:0051234	establishment of localization	1.00E-07	51
BP	GO:0016043	cellular component organization	2.10E-05	48
BP	GO:0046939	nucleotide phosphorylation	6.10E-05	5
BP	GO:0046031	ADP metabolic process	3.60E-05	5
BP	GO:0033036	macromolecule localization	1.10E-05	29
BP	GO:0006165	nucleoside diphosphate phosphorylation	6.10E-05	5
BP	GO:0071310	cellular response to organic substance	3.30E-05	24
BP	GO:0030154	cell differentiation	1.10E-05	32
BP	GO:1901700	response to oxygen-containing compound	2.50E-05	16
BP	GO:0009132	nucleoside diphosphate metabolic process	1.20E-05	6
BP	GO:0048738	cardiac muscle tissue development	3.30E-05	7
BP	GO:0010033	response to organic substance	3.60E-06	29
BP	GO:0033993	response to lipid	4.70E-05	11
BP	GO:0008104	protein localization	8.40E-06	26
BP	GO:0045184	establishment of protein localization	5.30E-07	23
BP	GO:0006810	transport	1.40E-07	50
BP	GO:0034284	response to monosaccharide	9.80E-05	5
BP	GO:0071396	cellular response to lipid	1.00E-04	9
BP	GO:0009135	purine nucleoside diphosphate metabolic process	4.80E-05	5
BP	GO:1901135	carbohydrate derivative metabolic process	1.80E-04	15
BP	GO:0009185	ribonucleoside diphosphate metabolic process	5.40E-05	5
BP	GO:0032526	response to retinoic acid	1.50E-04	4
BP	GO:0009746	response to hexose	9.80E-05	5
BP	GO:0071702	organic substance transport	1.90E-07	30
BP	GO:0015031	protein transport	8.20E-07	22
BP	GO:0071705	nitrogen compound transport	3.60E-07	26
BP	GO:0009179	purine ribonucleoside diphosphate metabolic process	4.80E-05	5
BP	GO:0009749	response to glucose	9.80E-05	5
BP	GO:0032020	ISG15-protein conjugation	1.10E-04	2
MF	GO:0042296	ISG15 transferase activity	1.10E-04	2
MF	GO:0005525	GTP binding	1.40E-05	13
MF	GO:0032561	guanyl ribonucleotide binding	3.70E-06	14
MF	GO:0036094	small molecule binding	6.50E-05	38
MF	GO:0005201	extracellular matrix structural constituent	1.00E-06	7
MF	GO:0019001	guanyl nucleotide binding	3.70E-06	14
MF	GO:0043167	ion binding	3.10E-05	61
CC	GO:0099512	supramolecular fiber	3.60E-05	13

Table S6. Enrichment Gene ontology for *Macronectes giganteus* specific gene families among 4 avian species.

GO Type	GO ID	Name	p-value
BP	GO:0003271	smoothened signaling pathway involved in regulation of secondary heart field cardioblast proliferation	5.68E-05
BP	GO:2000300	regulation of synaptic vesicle exocytosis	7.33E-05
BP	GO:0006511	ubiquitin-dependent protein catabolic process	1.36E-04
BP	GO:0010738	regulation of protein kinase A signaling	2.81E-04
BP	GO:0007283	spermatogenesis	3.41E-03
MF	GO:0004252	serine-type endopeptidase activity	3.03E-03
CC	GO:0005737	cytoplasm	1.20E-02

Table S7. Statistics of the *Macronectes giganteus* microsatellites.

Repeat Motif	Number of repeats								Total
	5	6	7	8	9	10	11–20	>21	
Dineucleotide									
AC/GT	5,124	1,731	699	357	205	133	257	5	8,511
AG/CT	4,250	942	408	186	98	59	135	19	6,097
AT/AT	2,749	965	444	212	131	75	147	10	4,733
CG/CG	74	22	12	4	2		1	0	115
Trinucleotide									
AGG	527	229	103	63	36	13	31	1	1,003
AGC	338	87	45	17	9	1	12	2	511
AAC	324	105	43	15	13	5	9	0	514
CCG	313	119	53	24	11	8	10	0	538
AAT	304	134	39	31	10	19	71	9	617
ACC	166	42	19	10	5	3	4	0	249
ATC	105	22	17	4	2	3	11	0	164
AAG	102	33	8	3	1	2	5	1	155
ACT	33	12	8	6	4		10	0	73
ACG	2	2		1	1		0	0	6
Tetranucleotide									
AAAC	258	85	22	1			0	0	366
AAAT	125	53	12	3	2		0	0	195
AAAG	38	18	4	3	1	1	7	0	72
AGGG	33	14	4	2	2		0	0	55
AAGG	21	10	8	6	1	3	9	0	58
ATCC	21	8	4	1			0	0	34
AGGC	18	2					0	0	20
AACC	17	5	1				0	0	23
ACAG	16	3	1				0	0	20
ACAT	14	5	2	1			0	0	22
Others	66	29	2	1	1	1	1	0	101
Pentanucleotide									
AAAAC	58	9	4	1	1	1	0	0	74
CCCGG	21	10	2	1			0	0	34
AAAAT	14	1	1	4	4	3	7	0	34
ACGGC	12	2	3	2		1	1	0	21
ACGGG	9	3					0	0	12
CCCCG	9	6		2			2	0	19
ATCCC	8	9	1	5	3	3	9	0	38
AAACC	7	2	1	2		1	0	0	13
AGGGC	6	2	2	1	1		0	0	12
AAGGG	2	1	1	1	1	2	6	17	31
Others	48	29	14	11	14	16	50	25	207
Hexa-nucleotide									
CCCCGG	12	10	1	2	3		1	0	29
ACCGGC	1	3	2	1			0	0	7
AAAGGG	1		2		1		1	1	6
AAAAAG		1					4	1	6
ATCCCC	1	2	1			1	0	0	5
AGGGGC	2	1	1			1	0	0	5
Others	29	11	15	2	3	6	14	2	82
Total	15,278	4,779	2,009	986	566	361	815	93	24,887

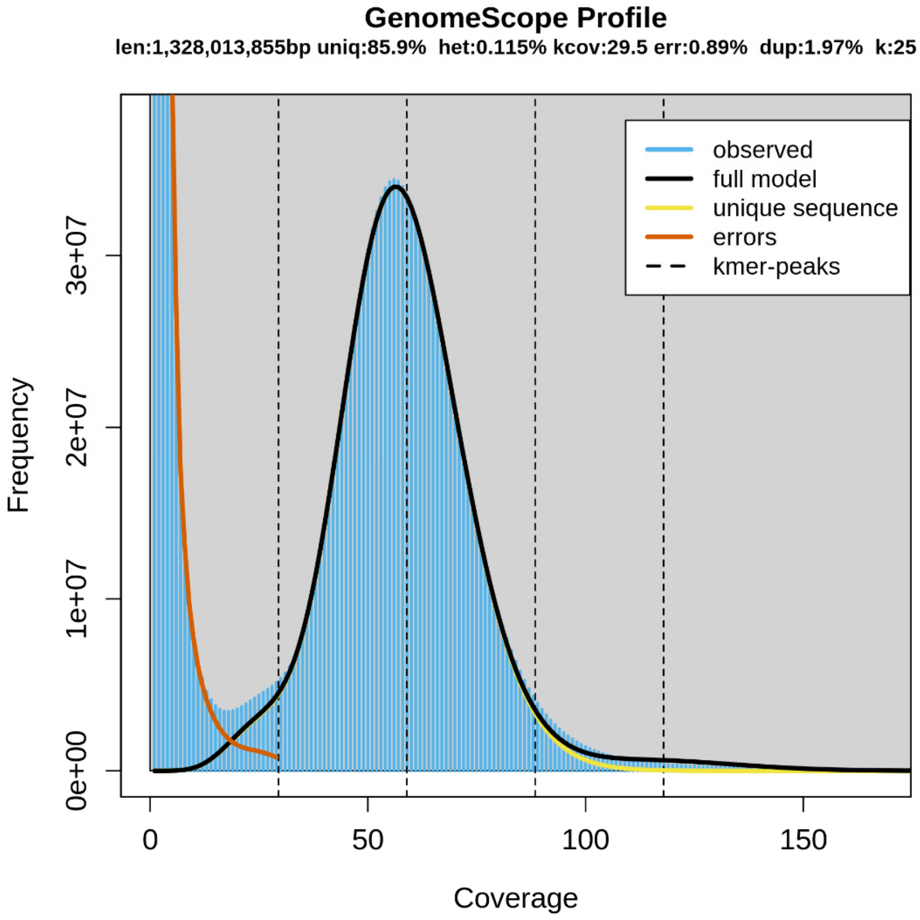

Figure S1. The histogram of the 25-mer depth distribution was plotted in GenomeScope to estimate genome size (1,328 Mb), heterozygosity level (0.115%).

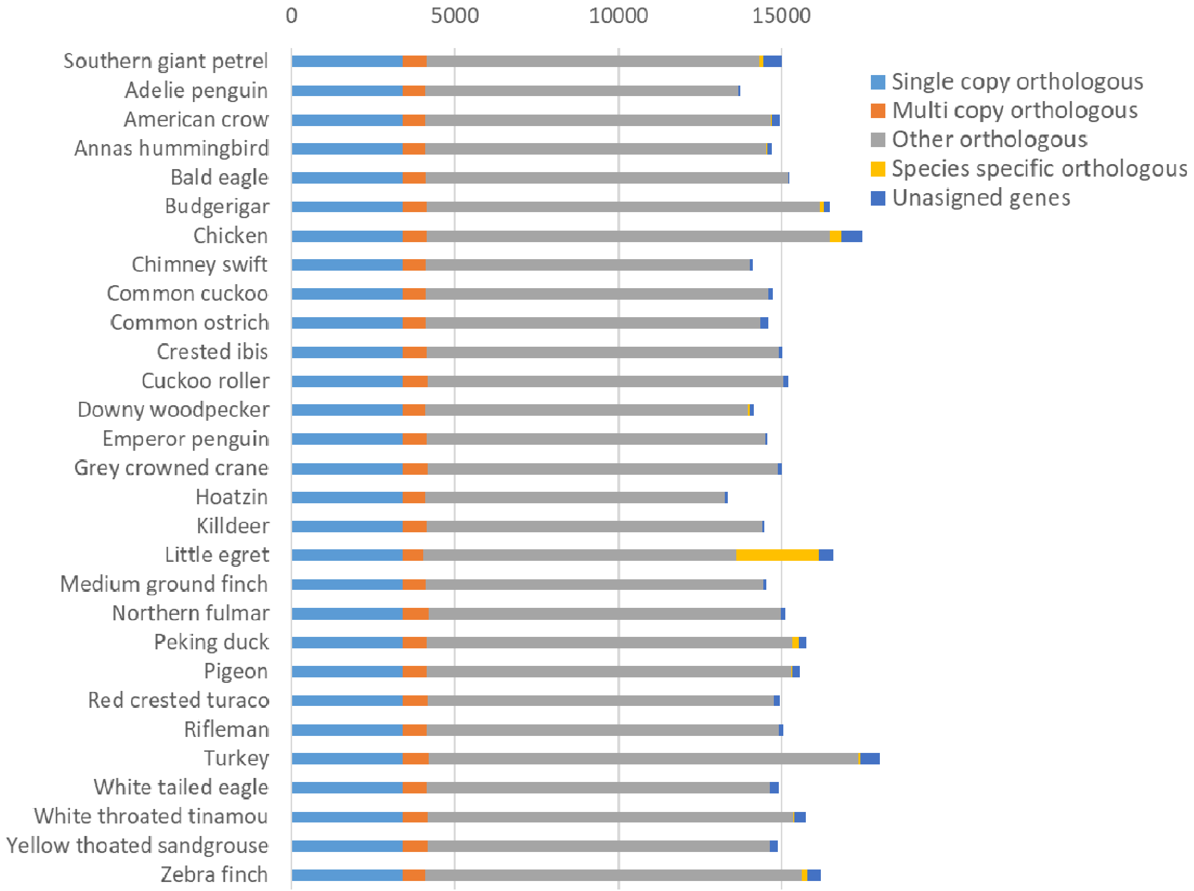

Figure S2. Comparison of avian orthologous genes. A comparative representation of orthologous and paralogous genes in 26 avian genomes are shown.

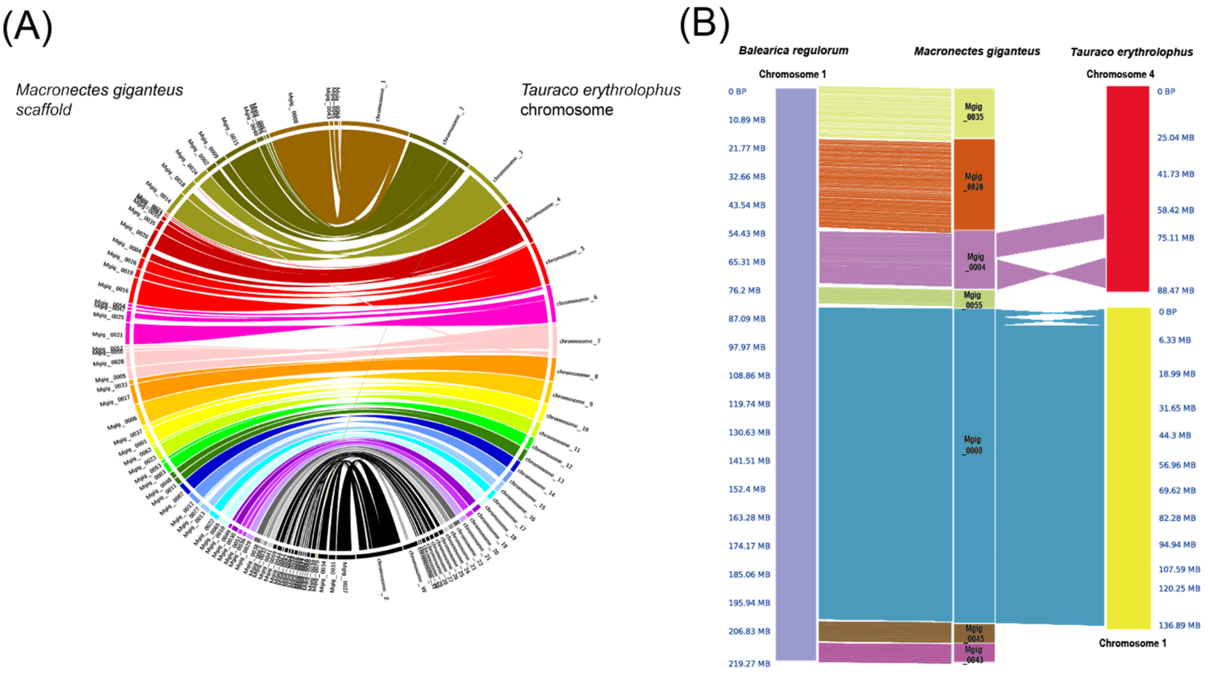

Figure S3. (A) Collinear relationship between *Macronectes giganteus* and *Tauraco erythrolophus*. Lines between the two rectangles show the shared syntenic blocks between the chromosomes, based on sequence homology, (B) Chromosome rearrangements between *M. giganteus* and *Balearica regulorum* and *Tauraco erythrolophus*.

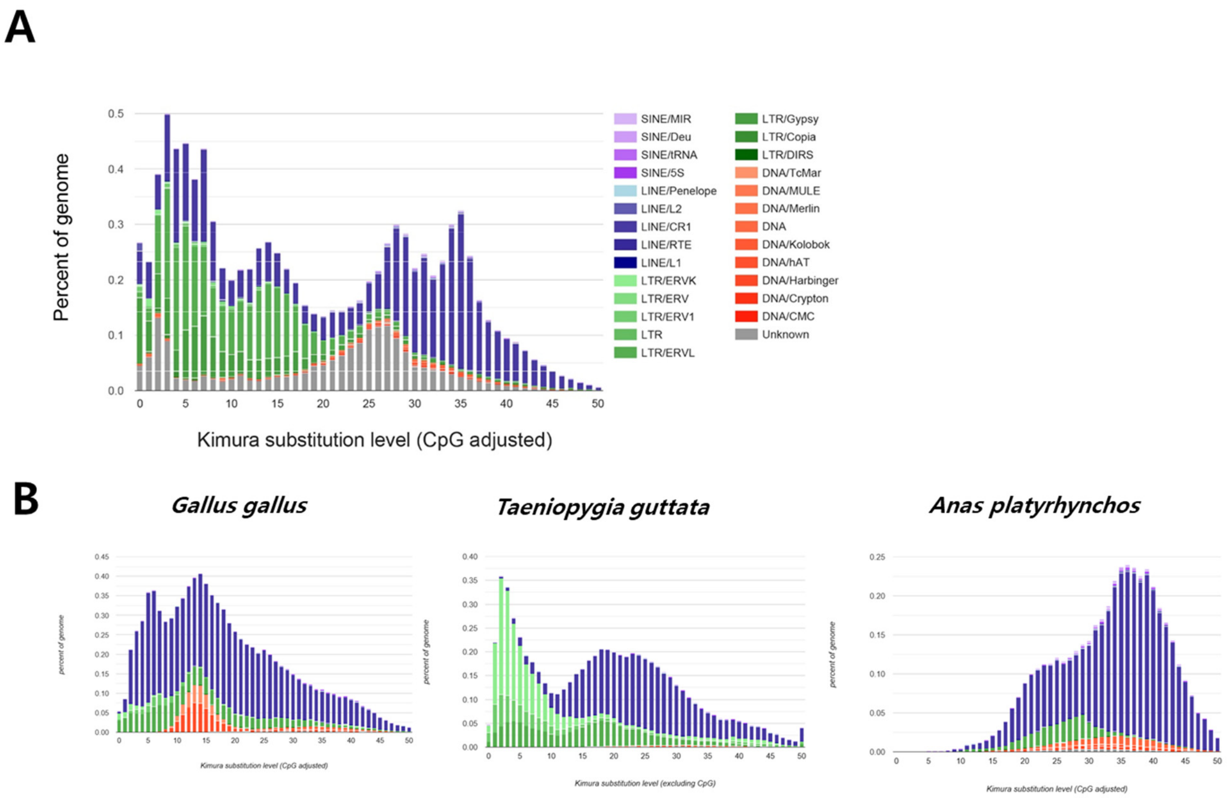

Figure S4. Interspersed Repeat Landscape of (A) *Macronectes giganteus* genome and (B) the interspersed repeat landscape of the Gallus gallus (galGal4), Taeniopygia guttata (taeGut1), and Anas platyrhynchos (anaPla1) genomes. Data from http://www.repeatmasker.org.

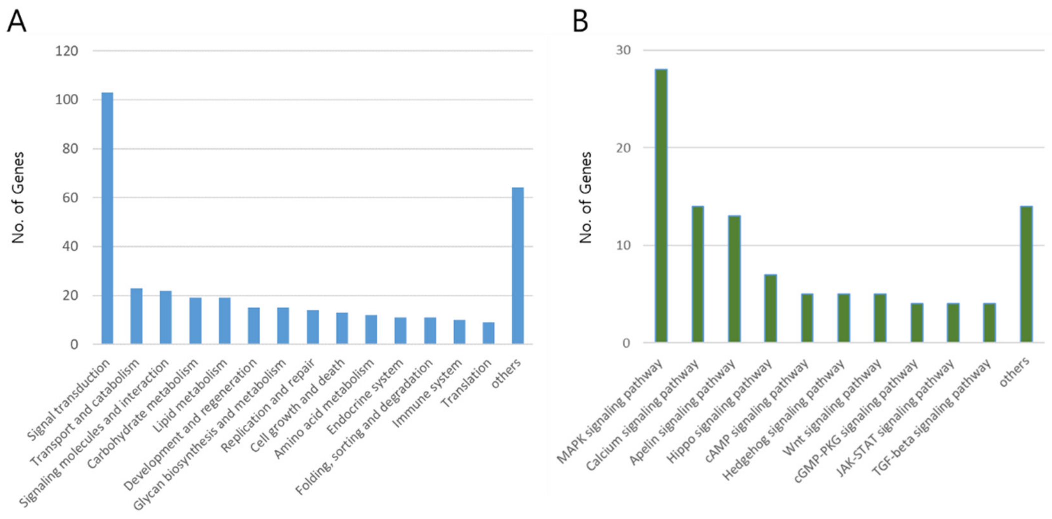

Figure S5. (A) KEGG pathway of expanded genes (B) Number of genes in the signal transduction pathway.

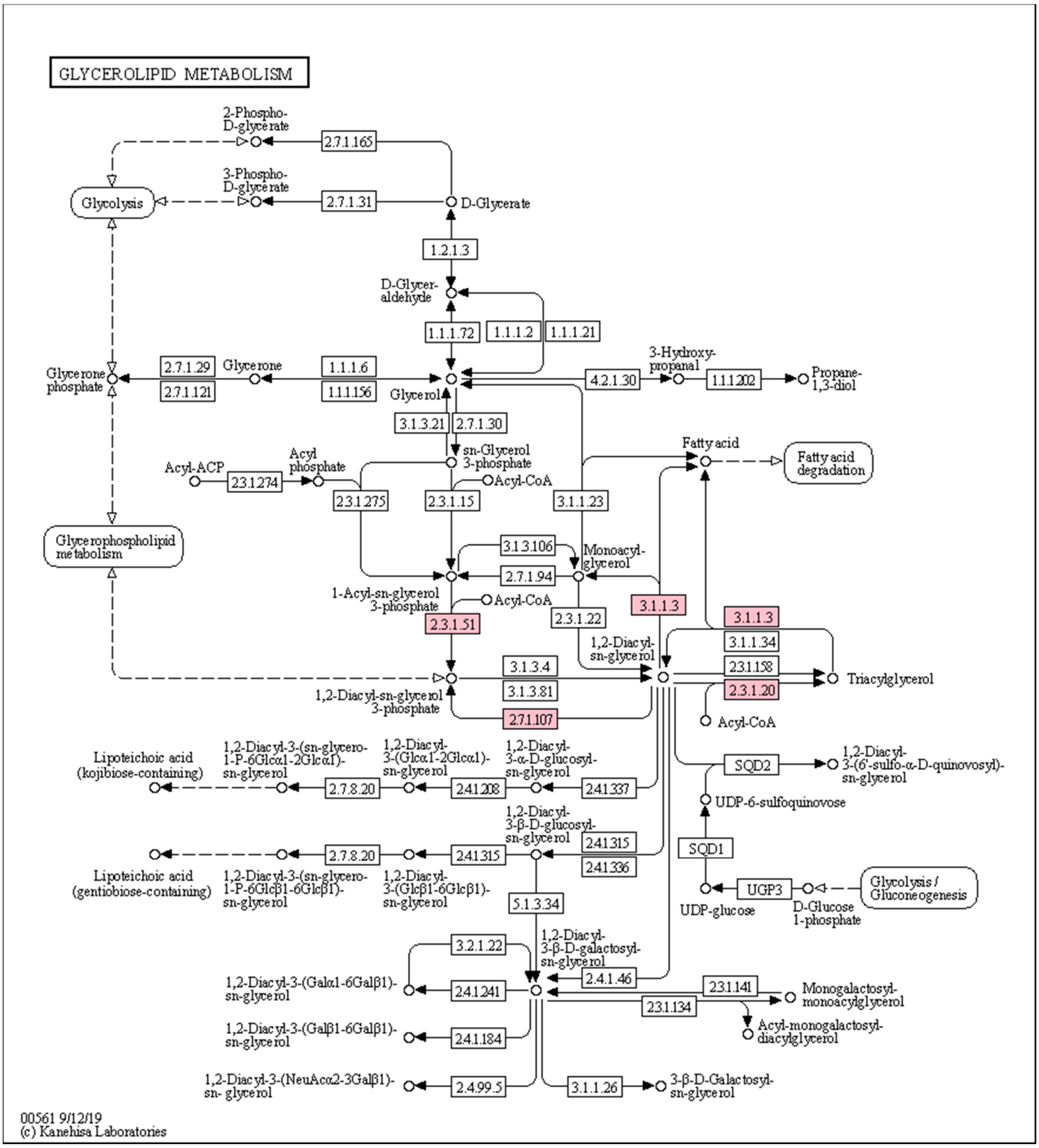

Figure S6. Expanded genes of glycerolipid metabolism in KEGG pathway. Red colour indicate expanded genes.
